# Decreased Copper in Alzheimer's Disease Brain Is Predominantly in the Soluble Extractable Fraction

**DOI:** 10.1155/2013/623241

**Published:** 2013-10-21

**Authors:** Alan Rembach, Dominic J. Hare, Monica Lind, Christopher J. Fowler, Robert A. Cherny, Catriona McLean, Ashley I. Bush, Colin L. Masters, Blaine R. Roberts

**Affiliations:** ^1^The Florey Institute of Neuroscience and Mental Health, The University of Melbourne, 30 Royal Parade Parkville, Parkville, VIC 3010, Australia; ^2^Elemental Bio-Imaging Facility, University of Technology, Broadway, Sydney, NSW 2007, Australia; ^3^Anatomical Pathology, The Alfred Hospital, Melbourne, VIC 3004, Australia

## Abstract

Alzheimer's disease (AD) is the leading cause of dementia and represents a significant burden on the global economy and society. The role of transition metals, in particular copper (Cu), in AD has become of significant interest due to the dyshomeostasis of these essential elements, which can impart profound effects on cell viability and neuronal function. We tested the hypothesis that there is a systemic perturbation in Cu compartmentalization in AD, within the brain as well as in the periphery, specifically within erythrocytes. Our results showed that the previously reported decrease in Cu within the human frontal cortex was confined to the soluble (*P* < 0.05) and total homogenate (*P* < 0.05) fractions. No differences were observed in Cu concentration in erythrocytes. Our data indicate that there is a brain specific alteration in Cu levels in AD localized to the soluble extracted material, which is not reflected in erythrocytes. Further studies using metalloproteomics approaches will be able to elucidate the metabolic mechanism(s) that results in the decreased brain Cu levels during the progression of AD.

## 1. Introduction

Alzheimer's disease (AD) is the predominant cause of dementia in the aging population and represents a mounting health epidemic [1]. Despite advances in understanding the events leading to the onset of cognitive decline, the principal cause of AD is still undetermined. The role of copper (Cu), iron (Fe), and zinc (Zn) in AD has become of significant interest because the dyshomeostasis of essential trace elements has been observed to have profound effects on cell viability and neuronal function [2, 3], which have been previously reviewed [4].

Cu, an essential element in the central nervous system (CNS), is crucial for life, but its unique redox propensity renders it toxic in circumstances of an increase pool of labile species [5–8]. Specific lesions in the Cu pathway can lead to a severe but treatable neurological impairment, including Menkes and Wilson's disease [9–11]. Cu displays a distinctly compartmentalized distribution throughout the brain, reflecting its diverse function in various neurological processes [12, 13]. 

Within the CNS, Cu is known to decrease in the frontal, occipital, and parietal lobes [14] amygdala and hippocampus in AD [15]. The process for this decline is not well understood, though extracellular plaques of aggregated amyloid-*β* (A*β*) are reported to be enriched with trace elements including Fe, Zn, and Cu [16]. Recently, it was also reported that frontal cortex from AD subjects had an increased propensity to bind exchangeable Cu, which correlated with oxidative damage observed in the tissue [17].

In cerebral spinal fluid (CSF), Cu levels are not observed at significantly different concentrations between AD and healthy controls (HC) [18–20]. However, within peripheral fluids, Cu dyshomeostasis has been more intensely studied. Reports of increased [19, 21], decreased [22, 23], or unchanged [24–26] serum or plasma Cu in AD have rendered total Cu levels too variable to be of diagnostic utility, for review see [27]. Yet, many studies have concluded that there is a subtle but consistent excess of nonbound or diffusible Cu in serum [21, 22, 28–34]. Despite this, a consensus has been thwarted by a lack of standardization and limitations driven by covariate influences on peripheral “high throughout” screening of Cu concentrations [35]. In other peripheral tissues, such as erythrocytes, superoxide dismutase 1 (SOD1) activity has been found to be diminished in AD [23]. This is thought to be due to a Cu deficiency in the enzyme, as reported previously [36].

In this study, we tested the hypothesis that there is a systemic perturbation in Cu compartmentalization in AD, within the frontal cortex as well as in the periphery, within erythrocytes.

## 2. Methods

### 2.1. Subjects

The AIBL study incorporates longitudinal neuroimaging, biomarker, neuropsychometric, and lifestyle data, see [37] for a detailed description of methods. Briefly, participants over the age of 65 years and fluent in English were divided into three groups; cognitively healthy individuals (HC), participants with mild cognitive impairment (MCI) based on the established criteria [38, 39], and participants diagnosed with *possible* or *probable* AD as defined by NINCDS-ADRDA criteria [40]. Written informed consent was obtained from all participants, and the study was approved by the appropriate institutional ethics committees.

### 2.2. Erythrocyte Preparation

Whole blood was collected from overnight fasted participants with a 27 g needle, into Sarstedt S-Monovette Lithium-Heparin 7.5 mL tubes (01.1608.100). The tubes were spun at 3,200 ×g for 30 min at room temperature, and the plasma was carefully taken off the hematocrit. The buffy coat was prepared by ficoll gradient centrifugation to extract the white blood cells. The erythrocytefraction was washed 3 times by adding 0.9% normal saline to an end volume of approximately 14 mL. Erythrocytes were dispersed by gently inverting the tubes 10 times and then centrifuged at 650 ×g for 10 minutes at 20°C with braking on. The final centrifugation was 1,500 ×g for 10 minutes at 20°C with braking on. The final saline wash was discarded, and the erythrocytes resuspended to an end volume of 6 mL in phosphate buffered saline (PBS) (pH 7.4), then aliquoted into polypropylene (Nunc cryobank, Denmark) tubes and snap-frozen in liquid nitrogen.

### 2.3. Fractionation of Brain Tissue for Biochemical Analysis

Brain tissues were obtained from the Victorian brain bank network, and all experiments were approved by the University of Melbourne health sciences, human ethics subcommittee (ID1136882). Hemisected frozen brains at −80°C were thawed to −20°C and sectioned in 1 cm slices. The meninges were removed from approximately 5 g of frontal cortex (Brodmann area 9), and the grey matter was dissected in to 0.2–0.5 g aliquots and stored at −80°C. The grey matter was allowed to thaw on ice and then homogenized using a BioMasher (Omni International). Tissue was placed in the BioMasher, the plunger was inserted, and then the apparatus was centrifuged at 100,000 ×g with a desktop centrifuge. After centrifugation, Tris buffered saline (TBS, 50 mM Tris pH 8.0, 150 mM NaCl) containing EDTA free protease inhibitors (Roche, 05056489001) was added at a ratio of 1 : 4 (tissue : buffer, w/v). The sample was then transferred to ultracentrifuge tubes and centrifuged at 100,000 ×g for 30 minutes at 4°C. The TBS supernatant, or “soluble” material, was collected and stored at −80°C before Western blot analysis. The pellet was resuspended in 100 mM NaCO_3_ pH 11.0 (1 : 4, tissue:buffer) and further centrifuged at 100,000 ×g for 30 minutes at 4°C. The supernatant, “peripheral membrane/vesicular” material was recovered, and the pellet was resuspended with 7 M urea, 2 M thiourea, 4% 3-[(3-cholamidopropyl)dimethylammonio]-1-propanesulfonate (CHAPS), 30 mM Bicine pH 8.5, and centrifuged at 100,000 ×g for 30 minutes at 4°C. The supernatant, “membrane” material, was recovered, and the resulting pellet was then incubated at room temperature with 70% formic acid for 16–18 hours before being centrifuged at 100,000 ×g for 30 minutes. After the sequential extraction, little to no observable material remained.

### 2.4. Induction Coupled Plasma-Mass Spectrometry (ICP-MS)

Frozen aliquots of erythrocytes or brain tissue homogenate fractions were thawed at room temperature. For brain homogenates, 50 *μ*L was diluted (1 : 20) with 950 *μ*L of 1% HNO_3_ (v/v). 50 *μ*L of washed erythrocytes were digested in equivalent volumes of concentrated (65%) HNO_3_ and H_2_O_2_ (Merck Millipore) at 80°C for 5 minutes, then diluted 1 : 20 with 1% HNO_3_. Cu concentration was determined using an Agilent Technologies 7700x ICP-MS system. The sample introduction system used a Teflon MiraMist parallel path nebulizer (Burgener Research Inc.) and standard Scott-type double-pass spray chamber (Glass Expansion). Helium was used as a collision gas. ICP-MS conditions were replicated from previously reported studies from our laboratory [35]. The instrument was calibrated using multielement standards (Accustandard, ICP-MS-2-1, ICP-MS-3, and ICP-MS-4; total of 44 elements) containing copper at 0, 5, 10, 50, and 100 ppb with ^89^Y as the internal standard for all isotopes of Cu. Interday relative standard deviations were determined using a quality control serum (Seronorm) with a certified copper level (84.55 *μ*g/L 95% CI 80.35–88.75 *μ*g/L) and were consistently between 2.0–5.0%. 

### 2.5. Statistical Analysis

Statistical analyses were performed with Prism version 6.0 (Graphpad Inc). To compare differences between the groups, a one-way ANOVA Bonferroni's multiple comparison test was used. Significant *P* values were <0.05.

## 3. Results

**Figure 1:**
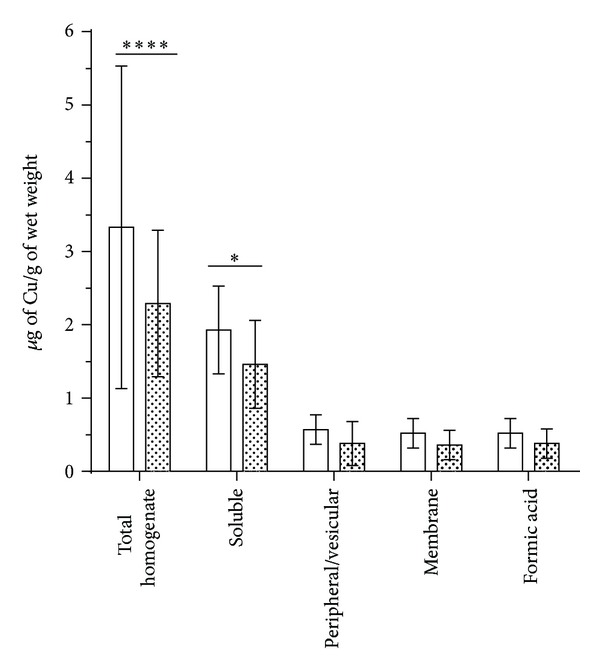
Cu content in human brain. Illustrates a significant decrease in total Cu in the soluble fraction of the extracted brain tissue. *P < 0.05, ****P < 0.0001; one-way ANOVA with Bonferroni's multiple comparison post hoc test of log transformed data. Cu is decreased in AD frontal cortex. Copper is significantly decreased in the total homogenate and soluble extracted material (*P* < 0.05). The only fraction that had a significant decrease was the soluble fraction indicating that the decrease in Cu observed in the total homogenate is localized to changes in the soluble fraction. HC *Healthy Control *(clear boxes), AD *Alzheimer's disease *(filled boxes).

**Figure 2:**
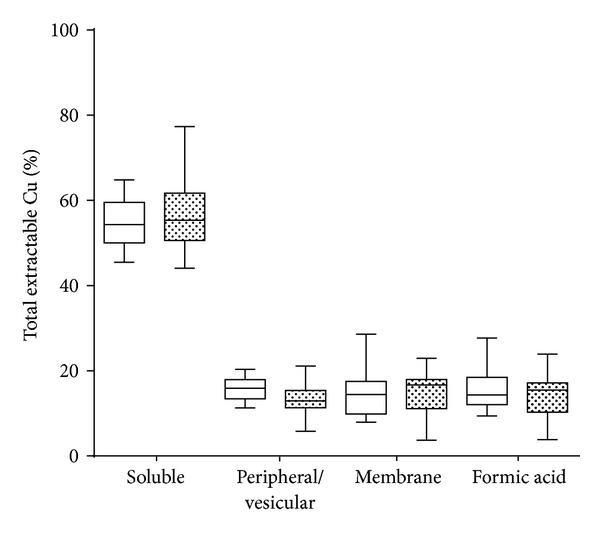
Cu content in human brain expressed as percentage distribution. The percent distribution of Cu extracted from human frontal cortex in the brain is conserved in AD and HC (total pooled). Box and whisker plots show the range, interquartile range, and median values. No significant difference was observed in the percentage of Cu in each of the corresponding fractions. Between 50–60% of the total Cu in human brain tissue is extractable in the soluble phase. No significant difference was observed between HC (clear boxes) and AD (filled boxes).

Table 1 and Figure 1 show that changes in Cu within the human frontal cortex were localized to the soluble fraction. We observed a significant decrease in total Cu levels consistent with previous studies [14, 15]. To investigate if the change in Cu was global or localized to a specific cellular compartment, we fractionated the brain tissue into four groups: soluble, peripheral membrane, and vesicular material, integral membrane and formic acid extractable material that contains predominantly insoluble plaques [41]. We only observed a significant decrease in Cu in the soluble fraction between AD and HC (Figure 1, *P* < 0.05, one-way ANOVA Bonferroni's multiple comparison test).  Table 2 shows the demography for the postmortem brain samples. Although we did observe a decreasing trend for Cu in each, we found that when Cu is expressed as a percentage of total Cu for each individual (Figure 2), no significant differences were observed, suggesting a conservation of Cu equilibrium that may be homoeostatically regulated. We observed that 50–60% of total tissue Cu content was localized to the soluble extractable material (Figure 2).

**Figure 3:**
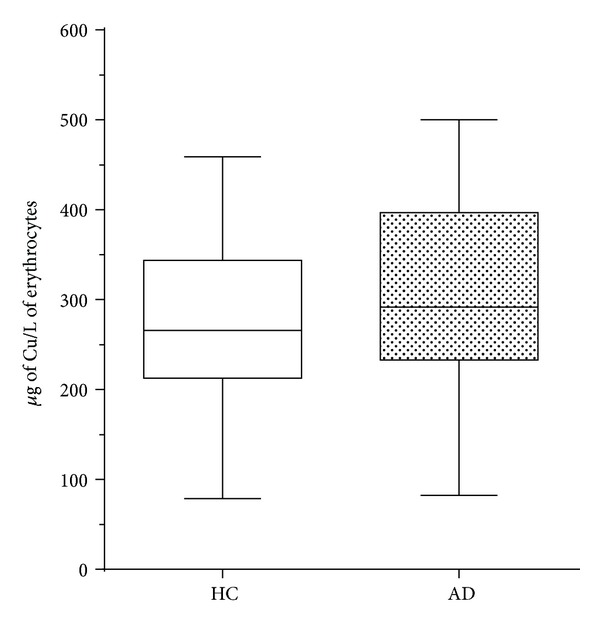
Cu concentration in human erythrocytes. The total Cu content of erythrocytes was determined using ICP-MS. No significant change (*P* = 0.53) in the level of Cu in red blood cells was observed between HC (*N* = 40) (clear boxes) and AD (*N* = 40) (filled boxes).

Previous studies have associated AD specific changes in erythrocytes [42, 43], including changes in the Cu dependent enzyme Cu, Zn-superoxide dismutase 1 (SOD1) [23]. We used well-characterized samples from the AIBL study to investigate the level of Cu in erythrocytes.  Table 3 shows the AIBL cohort demographics for individuals analysed for erythrocyte Cu concentrations.  Figure 3 shows that there was no significant difference in erythrocytic Cu concentration observed between AD and control samples.

## 4. Discussion

The aim of this study was to investigate the distribution and concentration of Cu in the frontal cortex and periphery of AD subjects when compared to age-matched healthy control samples. A number of studies have indicated that there is a significant perturbation in Cu coordination in AD within the frontal cortex [14, 17, 44] and periphery [3, 27]. Using well-characterized subjects, we were unable to demonstrate a significant difference in Cu levels within erythrocytes, a finding which is consistent with our investigations of serum Cu levels [35]. However, we did observe a significant difference within the frontal cortex, where AD tissue had significantly less Cu than controls (*P* = <0.0001). This observation is consistent with the frontal cortex having a unique susceptibility for Cu deficiency, compared to the periphery. Previous studies have used fractionation to investigate trace elements in AD brain [45], but data on changes to Cu concentrations is lacking. By fractionating brain tissue into several biochemical distinct subunits we observed that the decrease in Cu is mainly confined to the soluble fraction. The change in the soluble fraction is consistent with the reported deficiency of metallothioneins in the AD brain [46], though further metalloproteomic investigations are required to determine the extent that the Cu proteome is altered in AD neuropathology [47]. Surprisingly, no significant difference was observed in the formic acid fraction (Figure 1), contrary to our expectation that the AD plaques would demonstrate increased Cu in line with previous reports [48, 49]. The absence of an increase in Cu in the formic acid fraction highlights the importance of using spatially resolved techniques to measure tissue distribution of trace elements, including X-ray microfluorescence microscopy [50] and laser ablation ICP-MS [51, 52].

We have shown that over 50% of the Cu in human brain tissue can be extracted in the soluble or cytosolic portion of the homogenate. Further, the changes observed in the AD brain are due to specific changes in this soluble phase. As Cu has a strong propensity to participate in free radical chemistry, the distribution and delivery of Cu are carefully controlled by a set of Cu specific protein machinery [13, 53, 54]. This Cu handling system maintains less than one free Cu ion per cell [55]. Therefore, essentially all of the Cu is bound to biological ligands or is chaperoned by Cu regulatory proteins. Future investigations using a metalloproteomic approach [56–58] will be able to determine if the changes in the soluble Cu levels are specific to changes in binding partners, such as the reported decrease in metallothionein observed in AD [46]. As Cu is tightly regulated and exists as a ligated entity in the cell, it will be interesting to investigate if the change in soluble Cu are global, suggesting all Cu-proteins are decreased in AD, or are the changes restricted to selective proteins. In particular, detailed investigations of the stoichiometry of Cu proteins, like ceruloplasmin and the three reported metallothionein isoforms, will also be informative concerning Cu perturbations that may lead to deficiency in the CNS. 

In conclusion, this study has examined two pools of Cu in AD compared to age matched HCs. We showed that there is a specific change in the frontal cortex, indicating there is a perturbation in Cu homeostasis leading to a local diminution in concentration. We did not detect a significant difference in erythrocytes, suggesting that Cu disturbance may be confined to the brain in AD, precluding peripheral Cu levels as a useful biomarker for AD. The timing, systemic covariates, and mechanism(s) of this alteration still need to be systemically investigated, and advanced analytical metalloproteomic techniques will go a long way to answer these questions in the future.

## Figures and Tables

**Table 1:** Distribution of Cu in different cellular fractions and total levels from human brain.

Brain fraction	Cu (*μ*g/g of wet weight)		*P* value
	HC	AD	
Soluble	1.93 ± 0.6 (1.0–3.2)	1.46 ± 0.6 (0.6–3.3)	<**0.05**
Peripheral/vesicular	0.57 ± 0.2 (0.3–0.9)	0.38 ± 0.3 (0.7–1.1)	>0.05
Membrane	0.52 ± 0.2 (0.2–1.2)	0.36 ± 0.2 (0.05–0.8)	>0.05
Formic acid^¶^	0.52 ± 0.2 (0.3–1.0)	0.38 ± 0.2 (0.05–0.7)	>0.05
Total homogenate^§^	3.33 ± 2.2 (1.5–13)	2.29 ± 1.0 (0.9–4.7)	<**0.0001**

Concentration based on wet weight of tissue, mean ± standard deviation (range).

^¶^(HC) *N* = 20, (AD) *N* = 22, ^§^(HC) *N* = 24, and (AD) *N* = 23. Numbers in brackets are the 95% confidence intervals. *P* values were calculated using one-way ANOVA Bonferroni multiple comparison post hoc test. (NS: nonsignificant).

**Table 2:** Post-mortem subject demography.

	AD	HC	*P* value
	(*N* = 30)	(*N* = 27)	
Age (years)	78.0 (9.2)	77.0 (7.6)	>0.05
Gender females (%)	27	33	>0.05
*ApoE*ε*4 * carriers (%)	76	15	**<0.0001**
PMI (hours)	33.9 (22)	38.4 (14.3)	>0.05

Values are means (SD) unless noted above as otherwise. *P* values were calculated using *t*-test (two tailed). HC: healthy control, AD: Alzheimer's disease, *ApoE*ε*4*: Apolipoprotein E epsilon 4, PMI: postmortem interval.

**Table 3:** AIBL cohort demographics for individuals analysed for erythrocyte Cu levels.

	HC	AD	*P* value
	(*N* = 40)	(*N* = 40)	
Age (years)	76.8 (8.0)	77.3 (8.0)	>0.05
Gender females (%)	47.5	55	>0.05
*ApoE*ε*4* carriers (%)	37.5	60	<**0.05**
MMSE	28.4 (1.4)	18.1 (6.0)	<**0.0001**
CDR	0.075 (0.2)	6.175 (3.2)	<**0.0001**

Values are means (SD) unless noted above as otherwise. *P* values were calculated using one-way ANOVA Bonferroni multiple comparison post hoc test. HC: healthy control, AD: Alzheimer's disease, and MMSE: Mini-Mental State Examination. CDR: clinical dementia rating scale, *ApoE*ε*4*: Apolipoprotein E epsilon 4.

## References

[1] Thies W, Bleiler L (2012). 2012 Alzheimer’s disease facts and figures. *Alzheimer’s and Dementia*.

[2] Huang X, Moir RD, Tanzi RE, Bush AI, Rogers JT (2004). Redox-active metals, oxidative stress, and Alzheimer’s disease pathology. *Annals of the New York Academy of Sciences*.

[3] Squitti R (2012). Metals in alzheimer’s disease: a systemic perspective. *Frontiers in Bioscience*.

[4] Roberts BR, Ryan TM, Bush AI, Masters CL, Duce JA (2012). The role of metallobiology and amyloid-*β* peptides in Alzheimer’s disease. *Journal of Neurochemistry*.

[5] Massie HR, Aiello VR, Iodice AA (1979). Changes with age in copper and superoxide dismutase levels in brains of C57BL/6J mice. *Mechanisms of Ageing and Development*.

[6] Maynard CJ, Cappai R, Volitakis I (2002). Overexpression of Alzheimer’s disease amyloid-*β* opposes the age-dependent elevations of brain copper and iron. *Journal of Biological Chemistry*.

[7] Takahashi S, Takahashi I, Sato H, Kubota Y, Yoshida S, Muramatsu Y (2001). Age-related changes in the concentrations of major and trace elements in the brain of rats and mice. *Biological Trace Element Research*.

[8] Gaggelli E, Kozlowski H, Valensin D, Valensin G (2006). Copper homeostasis and neurodegenerative disorders (Alzheimer’s, prion, and Parkinson’s diseases and amyotrophic lateral sclerosis). *Chemical Reviews*.

[9] Mercer JFB, Livingston J, Hall B (1993). Isolation of partial candidate gene for Menkes disease by positional cloning. *Nature Genetics*.

[10] Gouider-Khouja N (2009). Wilson’s disease. *Parkinsonism and Related Disorders*.

[11] Waggoner DJ, Bartnikas TB, Gitlin JD (1999). The role of copper in neurodegenerative disease. *Neurobiology of Disease*.

[12] Hare DJ, Lee JK, Beavis AD (2012). Three-dimensional atlas of iron, copper, and zinc in the mouse cerebrum and brainstem. *Analytical Chemistry*.

[13] Davies KM, Hare DJ, Cottam V (2013). Localization of copper and copper transporters in the human brain. *Metallomics*.

[14] Plantin LO, Lying-Tunell U, Kristensson K (1987). Trace elements in the human central nervous system studied with neutron activation analysis. *Biological Trace Element Research*.

[15] Deibel MA, Ehmann WD, Markesbery WR (1996). Copper, iron, and zinc imbalances in severely degenerated brain regions in Alzheimer’s disease: possible relation to oxidative stress. *Journal of the Neurological Sciences*.

[16] Tiiman A, Palumaa P, Tougu V (2013). The missing link in the amyloid cascade of Alzheimer's disease—metal ions. *Neurochemistry International*.

[17] James SA, Volitakis I, Adlard PA (2012). Elevated labile Cu is associated with oxidative pathology in Alzheimer disease. *Free Radical Biology and Medicine*.

[18] Capo CR, Arciello M, Squitti R (2008). Features of ceruloplasmin in the cerebrospinal fluid of Alzheimer’s disease patients. *BioMetals*.

[19] Bucossi S, Ventriglia M, Panetta V (2011). Copper in alzheimer’s disease: a meta-analysis of serum,plasma, and cerebrospinal fluid studies. *Journal of Alzheimer’s Disease*.

[20] Bucossi S, Ventriglia M, Panetta V (2011). Copper in alzheimer’s disease: a meta-analysis of serum,plasma, and cerebrospinal fluid studies. *Journal of Alzheimer’s Disease*.

[21] Squitti R, Lupoi D, Pasqualetti P (2002). Elevation of serum copper levels in Alzheimer’s disease. *Neurology*.

[22] Brewer GJ, Kanzer SH, Zimmerman EA, Celmins DF, Heckman SM, Dick R (2010). Copper and ceruloplasmin abnormalities in Alzheimers disease. *American Journal of Alzheimer’s Disease and other Dementias*.

[23] Vural H, Demirin H, Kara Y, Eren I, Delibas N (2010). Alterations of plasma magnesium, copper, zinc, iron and selenium concentrations and some related erythrocyte antioxidant enzyme activities in patients with Alzheimer’s disease. *Journal of Trace Elements in Medicine and Biology*.

[24] Molina JA, Jiménez-Jiménez FJ, Aguilar MV (1998). Cerebrospinal fluid levels of transition metals in patients with Alzheimer’s disease. *Journal of Neural Transmission*.

[25] Ozcankaya R, Delibas N (2002). Malondialdehyde, superoxide dismutase, melatonin, iron, copper, and zinc blood concentrations in patients with Alzheimer disease: cross-sectional study. *Croatian Medical Journal*.

[26] Sedighi BS, Shariati M (2006). A study of serum copper and ceruloplasmin in Alzheimer’s disease in Kerman, Iran. *Neurology Asia*.

[27] Squitti R (2012). Copper dysfunction in Alzheimer's disease: from meta-analysis of biochemical studies to new insight into genetics. *Journal of Trace Elements in Medicine and Biology*.

[28] Squitti R, Ghidoni R, Scrascia F (2011). Free copper distinguishes mild cognitive impairment subjects from healthy elderly individuals. *Journal of Alzheimer’s Disease*.

[29] Squitti R, Pasqualetti P, Cassetta E (2003). Elevation of serum copper levels discriminates Alzheimer’s disease from vascular dementia. *Neurology*.

[30] Squitti R, Cassetta E, Dal Forno G (2004). Copper perturbation in 2 monozygotic twins discordant for degree of cognitive impairment. *Archives of Neurology*.

[31] Squitti R, Pasqualetti P, Dal Forno G (2005). Excess of serum copper not related to ceruloplasmin in Alzheimer disease. *Neurology*.

[32] Squitti R, Barbati G, Rossi L (2006). Excess of nonceruloplasmin serum copper in AD correlates with MMSE, CSF *β*-amyloid, and h-tau. *Neurology*.

[33] Squitti R, Bressi F, Pasqualetti P (2009). Longitudinal prognostic value of serum “free” copper in patients with Alzheimer disease. *Neurology*.

[34] Salustri C, Squitti R, Zappasodi F (2010). Oxidative stress and brain glutamate-mediated excitability in depressed patients. *Journal of Affective Disorders*.

[35] Rembach A, Doecke JD, Roberts BR (2013). Longitudinal analysis of serum copper and ceruloplasmin in Alzheimer's disease. *Journal of Alzheimer's Disease*.

[36] Klevay LM (2008). Alzheimer’s disease as copper deficiency. *Medical Hypotheses*.

[37] Ellis KA, Bush AI, Darby D (2009). The Australian Imaging, Biomarkers and Lifestyle (AIBL) study of aging: methodology and baseline characteristics of 1112 individuals recruited for a longitudinal study of Alzheimer’s disease. *International Psychogeriatrics*.

[38] Petersen RC, Smith GE, Waring SC, Ivnik RJ, Tangalos EG, Kokmen E (1999). Mild cognitive impairment: clinical characterization and outcome. *Archives of Neurology*.

[39] Winblad B, Palmer K, Kivipelto M (2004). Mild cognitive impairment—beyond controversies, towards a consensus: report of the International Working Group on Mild Cognitive Impairment. *Journal of Internal Medicine*.

[40] McKhann G, Drachman D, Folstein M (1984). Clinical diagnosis of Alzheimer’s disease: report of the NINCDS-ADRDA work group under the auspices of Department of Health and Human Services Task Force on Alzheimer’s disease. *Neurology*.

[41] Masters CL, Simms G, Weinman NA (1985). Amyloid plaque core protein in Alzheimer disease and Down syndrome. *Proceedings of the National Academy of Sciences of the United States of America*.

[42] Butterfield DA, Farmer BT, Markesbery WR (1985). Alzheimer’s disease: no alteration in the physical state of erythrocyte membrane glycoconjugates. *Annals of Neurology*.

[43] Mohanty JG, Shukla HD, Williamson JD, Launer LJ, Saxena S, Rifkind JM (2010). Alterations in the red blood cell membrane proteome in alzheimer’s subjects reflect disease-related changes and provide insight into altered cell morphology. *Proteome Science*.

[44] Schrag M, Mueller C, Oyoyo U, Smith MA, Kirsch WM (2011). Iron, zinc and copper in the Alzheimer’s disease brain: a quantitative meta-analysis. Some insight on the influence of citation bias on scientific opinion. *Progress in Neurobiology*.

[45] Wenstrup D, Ehmann WD, Markesbery WR (1990). Trace element imbalances in isolated subcellular fractions of Alzheimer’s disease brains. *Brain Research*.

[46] Uchida Y, Takio K, Titani K, Ihara Y, Tomonaga M (1991). The growth inhibitory factor that is deficient in the Alzheimer’s disease brain is a 68 amino acid metallothionein-like protein. *Neuron*.

[47] Richarz A-N, Brätter P (2002). Speciation analysis of trace elements in the brains of individuals with Alzheimer’s disease with special emphasis on metallothioneins. *Fresenius’ Journal of Analytical Chemistry*.

[48] Lovell MA, Robertson JD, Teesdale WJ, Campbell JL, Markesbery WR (1998). Copper, iron and zinc in Alzheimer’s disease senile plaques. *Journal of the Neurological Sciences*.

[49] Miller LM, Wang Q, Telivala TP, Smith RJ, Lanzirotti A, Miklossy J (2006). Synchrotron-based infrared and X-ray imaging shows focalized accumulation of Cu and Zn co-localized with *β*-amyloid deposits in Alzheimer’s disease. *Journal of Structural Biology*.

[50] James SA, De Jonge MD, Howard DL, Bush AI, Paterson D, Mccoll G (2013). Direct in vivo imaging of essential bioinorganics in Caenorhabditis elegans. *Metallomics*.

[51] Hare DJ, George JL, Grimm R (2010). Three-dimensional elemental bio-imaging of Fe, Zn, Cu, Mn and P in a 6-hydroxydopamine lesioned mouse brain. *Metallomics*.

[52] Hutchinson RW, Cox AG, McLeod CW (2005). Imaging and spatial distribution of *β*-amyloid peptide and metal ions in Alzheimer’s plaques by laser ablation-inductively coupled plasma-mass spectrometry. *Analytical Biochemistry*.

[53] Banci L, Bertini I, Ciofi-Baffoni S, Kozyreva T, Zovo K, Palumaa P (2010). Affinity gradients drive copper to cellular destinations. *Nature*.

[54] Thies W, Bleiler L (2011). 2011 Alzheimer’s disease facts and figures. *Alzheimer’s and Dementia*.

[55] Rae TD, Schmidt PJ, Pufahl RA, Culotta VC, O’Halloran TV (1999). Undetectable intracellular free copper: the requirement of a copper chaperone for superoxide dismutase. *Science*.

[56] Cvetkovic A, Menon AL, Thorgersen MP (2010). Microbial metalloproteomes are largely uncharacterized. *Nature*.

[57] Lancaster WA, Praissman JL, Poole FL (2011). A Computational framework for proteome-wide pursuit and prediction of metalloproteins using ICP-MS and MS/MS data. *BMC Bioinformatics*.

[58] Lothian A, Hare DJ, Grimm R, Ryan TM, Masters CL, Roberts BR (2013). Metalloproteomics: principles, challenges and applications to neurodegeneration. *Frontiers in Aging Neuroscience*.

